# Hepatic Arterial Embolization for the Treatment of Metastatic Neuroendocrine Tumors

**DOI:** 10.1155/2012/471203

**Published:** 2012-01-29

**Authors:** Eric Lee, H. Leon Pachter, Umut Sarpel

**Affiliations:** Surgical Oncology, Bellevue Hospital Center, 550 First Avenue, NBV 15 South 11, New York, NY 10016, USA

## Abstract

Neuroendocrine tumors (NETs) have a high predilection for metastasizing to the liver and can cause severe debilitating symptoms adversely affecting quality of life. Although surgery remains the treatment of choice, many liver metastases are inoperable at presentation. Hepatic arterial embolization procedures take advantage of the arterial supply of NET metastases. The goals of these therapies are twofold: to increase overall survival by stabilizing tumor growth, and to reduce the morbidity in symptomatic patients. Patients treated with hepatic arterial embolization demonstrate longer progression-free survival and have 5-year survival rates of nearly 30%. The safety of repeat embolizations has also been proven in the setting of recurrent symptoms or progression of the disease. Despite not being curative, hepatic arterial embolization should be used in the management of NETs with liver metastases. Long-term survival is not uncommon, making aggressive palliation of symptoms an important component of treatment.

## 1. Introduction

Neuroendocrine tumors (NETs) consist of a heterogeneous group of neoplasms of varying presentation and prognosis. While a complete list of this family of tumors includes dozens of distinct histopathologic subtypes from multiple different organ systems, the majority of NETs are carcinoid tumors of the gastrointestinal tract and endocrine tumors of the pancreatic islet cells [1–3]. Primary liver NETs have been reported but are unusual and will not be discussed in this paper [4].

NETs are relatively rare, with an incidence ranging from 2.5 to 5.3 per 100,000 [3, 5]. The prevalence is significantly higher at about 35 per 100,000, indicating that many patients are alive with disease [3, 6]. However, these figures may not capture the full burden of NET disease, since conflicting nomenclature systems exist, making them difficult to classify and quantify [1, 3, 5–7]. Although some NETs are more aggressive in their behavior than others, all have the potential for distant metastases and should be considered malignant. In patients with resectable tumors without metastatic disease, surgery is considered the gold standard and is the only curative option. 5-year survival rates in patients with localized, nonmetastatic NETs undergoing curative resection range from 80% to 100% [2, 6].

While many NETs are nonfunctioning, these tumors are traditionally categorized by their classic patterns of symptoms arising from the secretion of various peptides and hormones [8]. For example, patients with gastrin hypersecretion from a gastrinoma tumor may present with severe peptic ulcer disease refractory to treatment. Insulinomas can cause severe hypoglycemia, while glucagonomas manifest with hyperglycemia and diabetes. Other NETs include VIPoma, characterized by diarrhea and hypokalemia, and somatostatinoma, presenting with cholelithiasis, diabetes, and steatorrhea. Carcinoid tumors of the GI tract frequently produce serotonin (5-HT), which can manifest as skin flushing, severe diarrhea, abdominal cramping, and electrolyte abnormalities [8].

The symptoms associated with functional NETs may be severe and debilitating and detract significantly from the quality of life of the patient; therefore, aggressive treatments to reduce symptoms have an important role in therapy [8]. Even in the setting of an unresectable primary tumor or widely metastatic disease, most NETs have an indolent course; 5–10-year survival with stage IV disease is not uncommon and makes the treatment of symptoms a fundamental component of patient care [3, 9–14].

## 2. Treatment of Metastatic Disease

Because the majority of NETs arise from the gastrointestinal tract and the pancreas, the liver is the most common site of metastases [15]. In patients with liver metastases, 75–80% will present with these metastases at the time of diagnosis (synchronous), while 20–25% of patients develop liver metastases during the course of treatment (metachronous) [8]. An estimated 80–90% of patients with liver metastases are inoperable at presentation [16]. Many primary NETs are small in size, and it is not unusual for the liver metastases to be of greater volume than the primary tumor. Given the high tumor burden often associated with metastases, symptoms can become significantly worse as the disease advances. In addition, many of the peptides and hormones produced by NETs are eliminated by metabolism through the liver. Therefore, it is only after liver metastases are present, and these compounds spill directly into the systemic circulation, that the phenotype of the tumor is fully expressed. As a result, symptom control can become increasingly important as metastases develop.

Surgical resection of hepatic metastases has been shown to reduce symptoms and is indicated for this purpose alone [2, 12]. In addition, some data indicate an improvement in overall survival as well, and therefore metastasectomy should be considered for resectable disease even in patients with nonfunctional tumors [10, 12, 17]. Meaningful improvements in symptom control and overall survival can be achieved even if complete resection of metastatic disease is not possible. However, available data suggest that debulking should only be considered if greater than 90% of the tumor burden can be resected [11, 18]. Two-step surgeries can be considered to increase resectability in bilobar disease [19].

While resection is preferred, excessive tumor bulk, tumor location, and other biological factors often preclude surgery. Even with resection, recurrence is common (50–60% at 5 years) and repeat hepatectomy may not be feasible [20, 21]. In these cases where resection is not possible or would not be tolerated by the patient's physiology, interventional radiology alternatives to surgery have been proposed, including radiofrequency ablation, hepatic arterial radioembolization with ^90^Y, and hepatic arterial bland or chemoembolization. The goals of these therapies are twofold: to increase overall survival by stabilizing tumor growth, and to reduce the morbidity in symptomatic patients.

**Figure 1:**
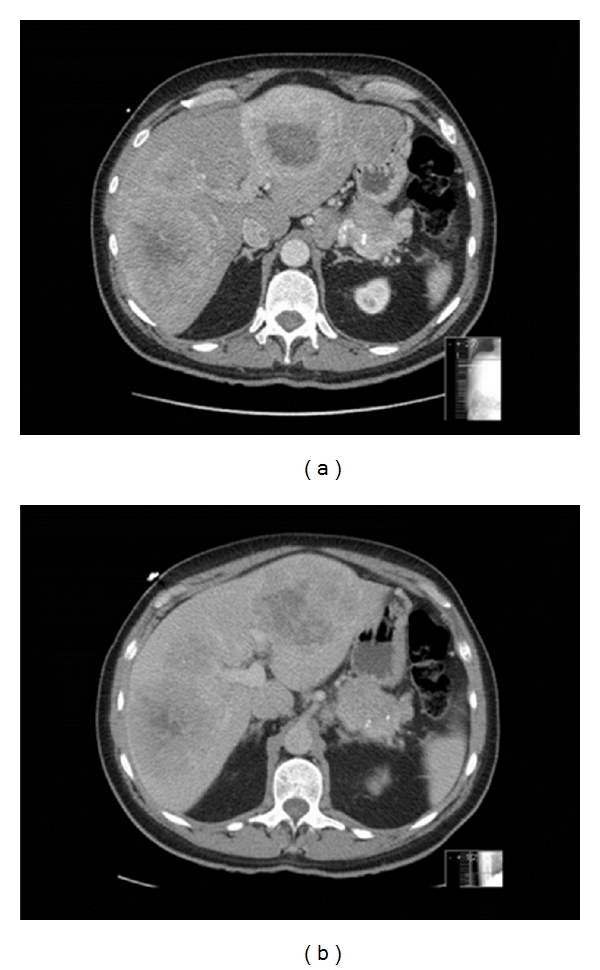
CT of bilobar hepatic metastases from a malignant NET in the (a) arterial phase and (b) venous phase. The characteristic enhancement of the tumor on arterial phase is apparent, as well as the relative darkening of the tumor on venous phase; the area of central necrosis is dark in both phases. Note the primary NET in the tail of the pancreas.

**Figure 2:**
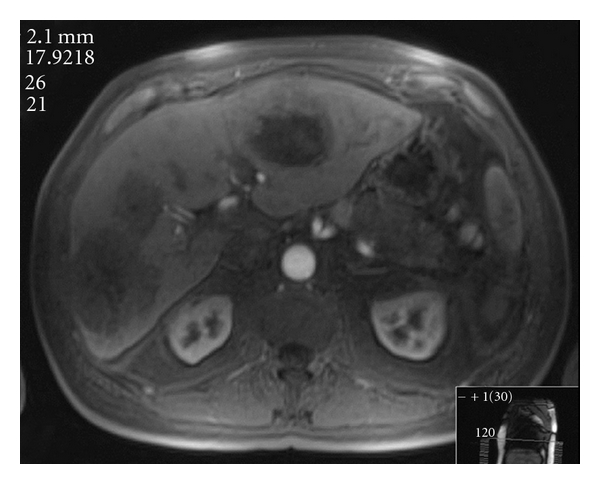
Post-TACE MR of bilobar hepatic metastases from the same patient in the arterial phase. Note the brightness of the aorta and lack of enhancement of the lesions compared to Figure 1, indicating the ischemia produced by the embolization.

The radiologic appearance of liver metastases from NETs is distinct and has important ramifications for treatment. Compared to liver metastases that are of gastrointestinal origin, metastases from NETs derive a greater amount of their blood supply from the hepatic artery. As a result, when imaged during the arterial phase, metastatic NETs will typically appear brighter than the surrounding liver; and during the venous phase when the normal liver parenchyma is filled with contrast, NET metastases will appear darker than the surrounding liver. In other words, NET metastases typically “light up and wash out” (Figure 1) [22]. This pattern of enhancement is similar to that seen with hepatocellular carcinoma and is ideally suited for the arterial embolization techniques more commonly associated with that disease. Treatment response can be assessed using radiographic measures by examining the degree of enhancement of the lesions following embolization procedures (Figure 2).

## 3. Indications and Contraindications

Since surgical metastasectomy is the most effective treatment, only patients with unresectable liver disease or who are unable to undergo surgery should be considered for embolization procedures. Previous resection of the primary tumor is not necessary, although the disease should be stable and not at risk for complications such as bleeding or obstruction [13].

Liver involvement greater than 75% is considered a relative contraindication to embolization, since these patients tend to have less response to treatment coupled with greater rates of complications [13, 23]. The presence of main portal vein thrombosis is a strict contraindication since hepatic arterial embolization relies upon the portal venous blood supply to rescue the nontumorous liver parenchyma. Therefore, hepatic arterial embolization in patients with complete portal vein thrombosis risks severe liver ischemia. 

Embolization in patients with bilirubin levels greater than 2-3 mg/dL has also been reported to be unsafe [13, 24, 25]. Even though the liver parenchyma is relatively spared with arterial embolization, there is nevertheless an ischemic insult that results in temporary liver insufficiency. Patients with already borderline liver function may be tipped over into frank liver failure following embolization. Accordingly, patients with ascites should be carefully considered for embolization procedures since its presence suggests poor liver function [13].

Finally, patients with general contraindications to angiography, intolerance of contrast media, peripheral vascular disease, or coagulopathies should not be considered for embolization.

## 4. Hepatic Arterial Embolization Technique

Occlusion of the hepatic artery causes selective ischemia to the tumor, while the remainder of the liver parenchyma is rescued by the portal venous flow. As a result, the tumor is disproportionately affected by the ischemic insult, with relative sparing of the normal parenchyma.

While not curative, hepatic arterial embolization procedures slow tumor growth and prolong progression-free survival, until the eventual revascularization from collateral angiogenesis resupplies the tumor. One lobe of the liver is treated per session to minimize the risk of liver failure [25]. If both lobes of the liver are involved with tumor, the contralateral side can be treated approximately one month after the initial embolization.

**Figure 3:**
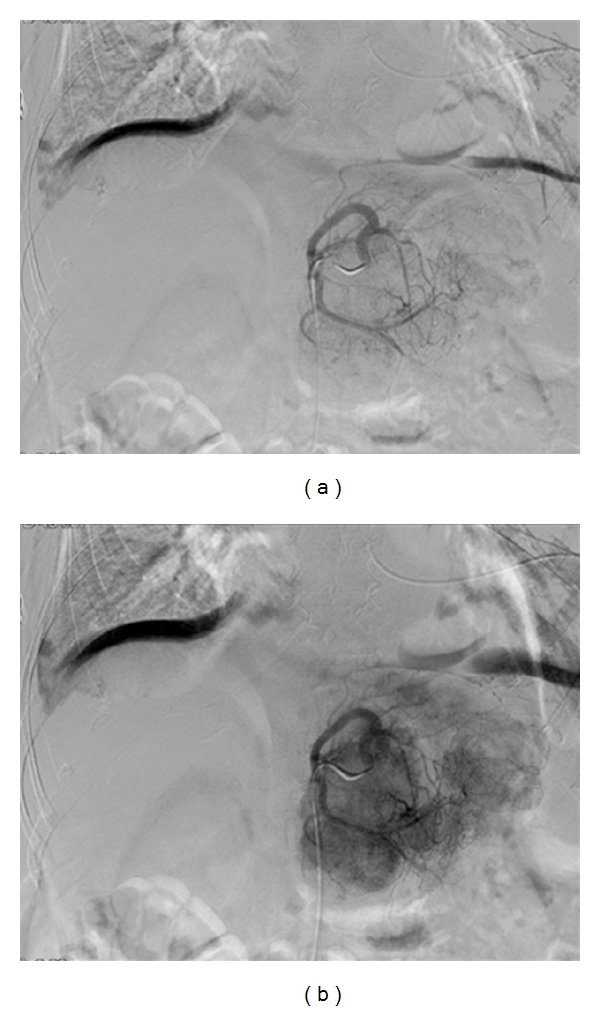
Arteriogram of the same patient with selective catheterization of the left hepatic artery from the femoral artery. Careful positioning of the catheter is important to minimize the risk of complications. Note the progressive tumor blush following the injection of contrast media.

Three types of hepatic arterial embolization techniques are currently in use: transarterial bland embolization (TAE), transarterial chemoembolization (TACE), and embolization using drug-eluting beads (DEB-TACE). These procedures all involve percutaneous access to the femoral artery, followed by selective cannulation of the hepatic artery and its derivatives to the affected lobe. Prior to embolization, an arteriogram is performed to identify the vascular anatomy supplying the tumor (Figure 3). If femoral access is not available, the brachial artery can be used as an alternative, although this route is more technically challenging. 

 In bland embolization, catheterization is typically followed by the injection of 50 *μ*m polyvinyl alcohol (PVA) particles, with or without ethiodized oil. These particles physically occludes blood flow through the selected hepatic artery, thereby inducing ischemic injury; if stasis remains unachieved, then larger 200–500-*μ*m PVA particles can be used [26–29]. Other embolic agents currently employed include gelfoam, cyanoacrylate, tris-acryl particles, and embospheres [26, 28, 29]. 

Previous studies established that TAE is effective at reducing tumor size as well as decreasing tumor hormone production for palliation of symptoms [11, 25–34]. Systemic adjuvant chemotherapy following TAE was noted to prolong the duration of symptom relief, prompting the development of embolization coupled with chemotherapy [35]. TACE combines the use of embolic material with an initial infusion of a chemotherapeutic agent. However, it is unclear whether the addition of intrahepatic chemotherapy improves the efficacy of embolization techniques.

The literature has not consistently shown a clear benefit of TACE over TAE, and no randomized head-to-head studies have been performed. While select reports have found that patients treated with TACE experienced slightly longer progression-free survival (PFS) and greater overall survival (OS), other reports have not found any benefit of TACE over TAE [28, 31, 32]. It is likely that the efficacy of these techniques is largely due to the ischemia produced by the embolization itself, with only secondary benefits derived from the addition of chemotherapeutic agents.

In addition, there is no consensus on which chemotherapeutic agents for TACE are the most efficacious in the treatment of liver metastases from NETs. Doxorubicin, mitomycin C, streptozocin, vinblastine, gemcitabine, fluorouracil (5-FU), and cisplatin have all been used for TACE, and some regimens employ them in combination. The most common regimen described in the literature is a three-drug combination of doxorubicin (20–30 mg), cisplatin (50 mg), and mitomycin C (10–30 mg) mixed with 10 mL ethiodized oil [13, 27, 36, 37]. Doxorubicin alone with ethiodized oil is the second most common regimen described [38–40]. Lipiodol is an oily agent which is typically used during TACE to enhance chemotherapy retention within the tumor [38]. Lipiodol appears bright white on CT imaging and therefore interferes with assessment of tumor viability. Surveillance following TACE should utilize MRI imaging since lipiodol does not appear on MRI images.

A third chemoembolization technique, DEB-TACE, uses 500–700 *μ*m embolic beads that are loaded with a chemotherapeutic drug, usually doxorubicin, which slowly elutes into the liver parenchyma over a period of 7–14 days [41–43]. The controlled release of chemotherapy allows for sustained, higher tumor levels of doxorubicin, while maintaining lower levels in the systemic circulation, which may decrease the incidence of systemic side effects. Studies have shown the OS and PFS in patients undergoing chemoembolization with DEB-TACE to be similar to TAE and TACE [41, 42].

Currently, all three techniques are actively in use, with no clear evidence for superiority of one approach over another.

## 5. Outcomes

Although there is a considerable amount of literature on hepatic arterial embolization treatments for metastatic NETs, most are retrospective reviews of smaller case series with historical controls; there are no randomized controlled trials. Due to the rare nature of NETs, prospective studies have not been feasible. Importantly, all methods of embolization have greater PFS and OS than no treatment or systemic chemotherapy alone [6, 11]. Most series include patients with both metastatic carcinoid tumors as well as pancreatic islet cell tumors in their cohorts. Patients with carcinoid NETs in general have longer OS and higher response rates than patients with pancreatic NETs, and as a result, outcomes should be interpreted accordingly [31, 32, 35].

One of the largest trials to date on the effects of embolization procedures is an analysis from Bloomston and colleagues on the outcomes of patients with metastatic carcinoid tumors undergoing TACE [36]. A cohort of 122 patients underwent 156 TACE procedures using a combination of doxorubicin (30 mg), mitomycin C (30 mg), and cisplatin (50 mg). The origin of the primary tumor was predominantly in the small bowel (47%), pancreas (21%), or lung (8%), with 14% of unknown origin. 81% of the patients presented with carcinoid syndrome, and the primary tumor had been previously resected in 75% of the patients. Interestingly, resection of the primary tumor did not prove to be predictive of survival. Following TACE, regression or stabilization of the hepatic metastases was observed in 94% of the patients, with a median duration of 19 months. Symptom improvement was reported in 92% of patients and was associated with a benefit in OS (41 months versus 8 months). Additionally, a lack of symptom improvement was associated with lack of radiographic response. PFS for the entire cohort was 10 months, and OS was reported to be 33.3 months with a 5-year survival of 28% from the date of the first TACE procedure [36].

Another large series by Swärd et al. examined 213 bland TAE procedures in 107 patients with metastatic carcinoid tumors. 106 of the patients had resection of the primary tumor, as well as prophylactic cholecystectomy [33]. Repeat TAE was performed in the setting of progressive disease, as demonstrated by two consecutive CT scans at least 6 months apart and a two fold increase in urinary 5-HIAA, a metabolite of 5-HT. Plasma chromogranin A levels, a general marker for NETs, were also recorded. Symptomatic improvement following TAE was reported in 71% of the patients, and an OS of 56 months from the date of embolization was shown for the group. Importantly, Swärd et al. were able to demonstrate a relationship between biochemical markers and survival benefit. Compared to patients who demonstrated no reduction in urinary 5-HIAA, patients with greater than 50% reduction experienced a 6-month gain in estimated survival, with an additional 6-month gain if the reduction was increased to 75%. In addition, increases in liver enzymes or chromogranin A both significantly correlated with reduced survival [33].

A more recent study by Pitt et al. compared the outcomes of 100 patients with carcinoid or islet cell tumors undergoing either TACE (*n* = 49) or TAE (*n* = 51) [28]. Particle embolization was performed with PVA, gelfoam, or embospheres; the chemotherapeutic agents used in TACE were cisplatin, doxorubicin, and mitomycin C. Both cohorts were similar with respect to age, gender, tumor type, and tumor burden. 67% of the TACE cohort underwent resection of the primary tumor, compared with 49% in the TAE cohort, although this difference was not significant. Response rates were 86% in the TACE cohort and 83% in patients treated with TAE and were not statistically different. Median overall survival from the date of the first procedure was also similar between the TACE and TAE groups, at 25.5 months and 25.7 months, respectively. In addition, 5-year survival rates for the TACE and TAE groups were not statistically different at 19% and 13%, respectively. Furthermore, the TACE and TAE groups exhibited similar complication rates (2.4% versus 6.6%, resp.) and mortality rates (0.8% versus 1.8%, resp.). In contrast to the study by Bloomston et al., resection of the primary tumor was significantly associated with an increase in overall survival: 73 months versus 28 months, respectively, from the time of diagnosis of metastatic disease. No other factors were found to be significantly predictive of survival, including tumor type, tumor burden, embolization type, and resection of liver metastases [28].

Patients with extensive liver tumor burden experience poorer response to embolization, as well as a greater rate of major complications. A major complication rate of 29% following embolization has been reported in patients with large volume disease [23, 31]. However, hepatic arterial embolization can still be of benefit in this group of patients. Kamat et al. demonstrated a median overall survival of 17.9 months and a PFS of 9.2 months using either TAE or TACE in patients with greater than 75% liver involvement. While only 44% of the patients demonstrated radiologic response, 65% showed improvement of symptoms, indicating that symptom relief can be used to guide therapy irrespective of radiologic findings [23].

## 6. Repeated Embolization

Despite embolization, most patients will exhibit disease progression as determined through both radiologic measures and the resumption of symptoms. These patients should be strongly considered for repeat embolization. Repeat embolizations are generally spaced 4–6 weeks apart to allow for the liver to recover fully [33].

The outcomes of patients undergoing repeat TACE have been reported in the literature, including a study by Varker et al. [37]. Although both radiologic and symptomatic responses were found to be slightly lower than for patients having their initial TACE (61% versus 82% and 77% versus 92%, resp.), this did not reach statistical significance. In addition, OS and PFS were similar between patients having initial versus repeat TACE. Of note, patients undergoing repeat embolization better tolerated the procedure and had a lower complication rate (11% versus 23%) than patients undergoing initial embolization [37]. These results indicate that repeat TACE is safe and effective in patients with progressive disease after initial embolic therapy and should be aggressively pursued to maintain disease control. A summary of the outcomes can be found in Table 1.

## 7. Complications

Both minor and serious complications as defined by the Society of Interventional Radiology standard criteria [44] are not uncommon among patients undergoing embolization. A review of the literature has shown the incidence of serious complications to range from 3% to 17% in most series [11, 13, 17, 25–34, 36–38, 45].

Postembolization syndrome (fever, nausea, vomiting, abdominal pain, and elevated liver enzymes) has been found to occur in the majority of patients but typically subsides within three days post-procedurally [11, 16, 27, 29–32, 38, 40]. There are anecdotal reports that the occurrence of postembolization syndrome correlates with the amount of tumor insult and that a robust physical response to embolization is in fact a positive prognostic indicator [46].

Hepatic failure, hepatic abscess, hepatorenal syndrome, sepsis, and severe hypertension occurring during embolization can all result from the local ischemia induced by arterial embolization [11, 13, 16, 30, 31, 33, 36]. Patients with bilioenteric anastomoses or large tumors (greater than 5 cm) are especially at risk for hepatic abscess formation after embolization [47, 48]. Due to the risk of abscess formation, many physicians advise prophylactic antibiotic administration prior to the procedure [25, 27, 29, 36, 38]. Patients who develop a hepatic abscess can be treated with percutaneous drain placement and parenteral antibiotics; rarely liver resection may be indicated for persistence [30].

Cholecystitis and pancreatitis are both relatively common complications of hepatic arterial embolization. These events are thought to be due to reflux of embolic material into the cystic artery or pancreaticoduodenal artery respectively, causing ischemic injury to these organs. Careful positioning of the catheter tip into the intrahepatic portion of the hepatic artery, along with gentle infusion techniques, is thought to limit the incidence of these potentially serious complications [24].

Mortality following embolization procedures is rare in high-volume centers. In a study of 26 patients undergoing 62 TACE procedures, Kress et al. reported fatal hepatic failure in 2 patients (3.2%) within 30 days after embolization [38]. Similar 30-day mortality rates have been found by other investigators, ranging from 0% to 6%, the majority of which were caused by hepatic failure, acute renal failure, sepsis, and myocardial infarction [17, 27, 30, 31, 36].

As to be expected, both acute and chronic renal failure secondary to contrast media administration during arteriogram have been noted as a severe complication from TAE and TACE [23, 27, 38]. Finally, all hepatic arterial embolization procedures carry the potential complications which accompany femoral arterial catheterization, including groin hematoma, peripheral embolization, and arterial dissection [27, 30, 33].

Of note, patients having TAE have similar rates of complication compared to patients having TACE [28]. Additionally, DEB-TACE procedures have demonstrated comparable morbidity rates, with 30%–60% of patients experiencing elements of postembolization syndrome [42]. Both Gaur et al. and de Baere et al. reported mortality rates of 5% in patients undergoing DEB-TACE [41, 42]. Table 2 summarizes the characteristics of hepatic arterial embolization.

## 8. Conclusion

Patients with NETs metastatic to the liver are often afflicted with debilitating symptoms that severely affect quality of life, and most patients with NETs ultimately die from progression in the liver. As a result, control of the hepatic tumor burden should be the primary goal in the management of patients with metastatic NETs. Surgical metastasectomy is considered preferable, although there are no randomized controlled trials comparing resection to nonsurgical therapies. In cases where resection is not feasible, interventional radiologic therapies such as hepatic arterial embolization can be used to control disease progression. The characteristic arterial enhancement of NETs can be taken advantage of, allowing selective embolization of the tumor while sparing normal parenchyma. Several modalities and chemotherapy regimens exist, and all have proven efficacy. Although not curative, hepatic arterial embolization can improve symptoms and reduce or stabilize tumor progression, prolonging PFS and OS, and should be the mainstay of treatment of patients with liver metastases from NETs. Embolizations should be repeated as needed to control symptoms and slow tumor growth.

## Figures and Tables

**Table 1:** Outcomes of hepatic arterial embolization in large published case series.

** Author**	**Type of embolization**	**No. of patients/No. of embolization procedures**	**Survival**	**Comments**
Bloomston et al. [36]	TACE	122/156	PFS: 10 months OS: 33 months 5-yr survival: 28%	Symptom improvement associated with increase in OS

Swärd et al. [33]	TAE	107/213	OS: 56 months	Increased survival with reduction in 5-HIAA; reduced survival with increased AST or chromogranin A

Pitt et al. [28]	TACE and TAE	100/229	TACE OS: 25.5 TAE OS: 25.7 TACE 5-yr survival: 19% TAE 5-yr survival: 13%	OS and 5-yr survival not statistically different between TAE and TACE; resection of primary tumor increased OS

Kamat et al. [23]	TACE and TAE	60/123	OS: 18 months PFS: 9 months	Patients had greater than 75% hepatic tumor burden; symptom improvement seen in 65%; major complication rate of 29%

Varker et al. [37]	Repeat TACE	27/54	OS: 28 months PFS: 5 months	Repeat TACE associated with similar OS and PFS, and lower complication rates compared to single TACE

**Table 2:** Characteristics of hepatic arterial embolization for NET metastases.

**Indications**	
Hepatic metastases of NET	
Nonoperative candidates	
Symptomatic and asymptomatic tumors	

**Contraindications**	
Main portal vein thrombosis	
Bilirubin greater than 2-3 mg/dL	
Hepatic tumor burden greater than 75%	
Contraindications to angiography	

**Outcomes**	
Mortality 0–6%	
Median OS 25–56 months	
5-yr survival 13–28%	

**Common chemotherapeutic agents**	
None (bland embolization)	
Doxorubicin	
Mitomycin C	
Cisplatin	

**Most frequent complications**	
Postembolization syndrome	
Hepatic abscess	
Hepatic failure	
Cholecystitis	
Pancreatitis	

**Indications for repeat embolization**	
Increase in tumor size or tumor enhancement	
Progression of symptoms	
